# Phytochemistry, Biological Synthesis, and Pharmacology of Flavonoids from Genus *Polygonatum*

**DOI:** 10.3390/molecules31101558

**Published:** 2026-05-07

**Authors:** Hong-Qun Liu, Zheng-Yi Qu, Bo He, Cai Shao, Jun Zhang, Wei Hou

**Affiliations:** 1 College of Medicine, Changchun Sci-Tech University, Changchun 130600, China; liuhongqun2014@163.com; 2 Institute of Special Wild Economic Animals and Plants, Chinese Academy of Agricultural Sciences, Changchun 130112, China; qu9581@163.com (Z.-Y.Q.);; 3 Jilin Provincial Key Laboratory of Traditional Chinese Medicinal Materials Cultivation and Propagation, Changchun 130112, China; 4 Key Laboratory of Se-Enriched Products Development and Quality Control, Ministry of Agriculture and Rural Affairs, Ankang 725000, China

**Keywords:** *Polygonatum* Mill., flavonoids, phytochemistry, biosynthesis, pharmacology

## Abstract

*Polygonatum* Mill. is a member of the Liliaceae family that is widely distributed across the world. Modern pharmacological and phytochemical studies have demonstrated that *Polygonatum* species have abundant bioactive chemical constituents, including saponins, flavonoids, and polysaccharides. Among them, flavonoids have attracted growing research interest due to their remarkable pharmacological properties; to date, 93 flavonoids have been isolated and characterized from this genus. These flavonoids exhibit a broad spectrum of pharmacological activities, including antioxidant, anti-diabetic, anticancer, anti-inflammatory, and antibacterial effects. For this review, articles were retrieved by searching online databases including ScienceDirect, Web of Science, Google Scholar and CNKI. Research published from 1997 to March 2026 is systematically reviewed herein, with a focus on the structural characteristics, plausible biosynthetic pathways and pharmacological activities of flavonoids from this genus. This review presents updated, comprehensive, and classified information on the phytochemistry and pharmacology of flavonoids in the genus *Polygonatum*, aiming to provide a reference for the further exploitation and utilization of *Polygonatum* resources.

## 1. Introduction

*Polygonatum*, a perennial herbaceous genus belonging to the Liliaceae family, is widely distributed in the northern temperate zone, including some regions of Asia and Europe, with approximately 79 species worldwide and 39 species in China [1]. Some species in the genus *Polygonatum* have been widely used in traditional Chinese medicine (TCM) and as a functional food in China and Southeast Asian countries for more than 2000 years. For example, the rhizome of *P. odoratum* is recorded as “Yuzhu”, whereas the rhizomes of *P. sibiricum*, *P. kingianum*, and *P. cyrtonema* are collectively referred to as “Huangjing” [2,3]. As typical yin-nourishing medicinal materials, these herbs exert anti-senescence effects and are widely applied for the management of osteoporosis, general debility, physical fatigue, diabetes and pulmonary diseases [4,5]. As one of the most important secondary metabolites in *Polygonatum*, flavonoids not only play a crucial role in the plant’s growth, development, and stress resistance but also serve as key bioactive components responsible for its medicinal and health-promoting properties, making them a focus of recent research in phytochemistry and pharmacology [6,7,8].

Flavonoids isolated in *Polygonatum* exhibit remarkable structural diversity, with representative subclasses including homoisoflavanones, flavones, isoflavones, chalcones, flavanones, isoflavanones, flavonols, and pterocarpans [9]. Among these, homoisoflavanones are an abundant and characteristic subclass, mainly distributed in *P. odoratum*, *P. kingianum*, *P. cyrtonema*, *P. sibiricum*, and *P. hunanense*, with *P. odoratum* containing the largest number of homoisoflavanones [10]. Structurally, homoisoflavanones in *Polygonatum* are distinguished by a unique C6-C4-C6 skeleton, while other subclasses, such as flavones, isoflavones, and flavonols, possess the classic C6-C3-C6 skeleton [11]. Notably, flavones in *Polygonatum* are mainly present in the form of C-glycosides, which are more stable than O-glycosides and contribute to the stability of these compounds in the plant [12]. The structural diversity of these flavonoids is closely related to their modification patterns, including hydroxyl substitution, methyl substitution, and glycosylation, which further affect their solubility, stability, and biological activities.

This review updates and summarizes the flavonoid constituents, biosynthetic pathways and pharmacological activities of *Polygonatum*. It aims to clarify the current research progress, highlight a variety of flavonoid monomers as well as their biological synthesis in this genus, and provide theoretical support and references for further investigations into the metabolic regulation, structural modification, and medicinal development and industrial application of *Polygonatum* plants.

## 2. Methodology

A comprehensive survey was performed on the published literature up to March 2026 concerning the chemical constituents, biosynthetic pathways, and pharmacological activities of flavonoids from the genus *Polygonatum*. The literature retrieval was performed using online databases including ScienceDirect, Web of Science, Google Scholar, PubMed, CNKI, Baidu Scholar, and other sources (such as the *Chinese Pharmacopoeia* 2020 edition, Flora of China). The search terms used for data collection were “*Polygonatum*”, “*Polygonatum* and flavonoids”, “*Polygonatum* and homoisoflavanones”, “*Polygonatum* and chemical constituents”, “Biosynthetic pathways of *Polygonatum* flavonoids”, “*Polygonatum* and pharmacological activities”, and “*Polygonatum* flavonoids and antioxidant activity”. A total of 100 publications published between 1997 and March 2026 were included in this study. Among them, 35 studies were related to chemical constituents, 8 studies were related to biosynthesis, 32 studies were related to pharmacological effects, and 25 studies were related to practical applications. Articles focused on cultivation techniques, extraction procedures, and content determination were excluded. Chemical structures were drawn using ChemDraw Professional (version 14.0).

## 3. Phytochemical Constituents

The genus *Polygonatum* is widely recognized as a rich source of structurally diverse flavonoid-type secondary metabolites [13,14]. Based on the comprehensive literature retrieval and screening, a total of 93 isolated flavonoid monomers have been documented and categorized into eight distinct classes: homoisoflavanones, flavones, isoflavones, chalcones, flavanones, isoflavanones, flavonols, and pterocarpan. Among these classes, homoisoflavanones represent the largest and most characteristic group, while the others serve as auxiliary components that collectively enhance the chemical diversity of *Polygonatum*. Each category possesses unique substitution patterns, stereochemical properties, and glycosylation modifications, all of which are summarized herein. Detailed information, including the chemical names, structures, formulas, origins, and relevant references, is provided in Table 1.

As a unique subclass of flavonoids, homoisoflavonoids exhibit limited natural distribution. To the best of our knowledge, only six plant families have been reported to contain this class of compounds, including Asparagaceae, Gentianaceae, Fabaceae, Polygonaceae, Orchidaceae and Portulacaceae [15]. Asparagaceae plants constitute the predominant botanical source of naturally occurring homoisoflavonoids. Consequently, *Polygonatum* plants are rich in homoisoflavonoids, and *P. odoratum* yields the most isolated homoisoflavonoid monomers to date. A total of 54 homoisoflavonoids feature the unique 3-benzylchroman-4-one structural skeleton, clearly distinguishing them from conventional flavonoids. Most possess a (3R) or (3S) chiral configuration at the C-3 position, and several exist as racemic mixtures. Their A-ring (5,7-dihydroxylated) is frequently modified by methyl and methoxyl groups at C-6 and/or C-8 positions. The benzyl side chain at C-3 commonly bears hydroxyl and methoxyl substituents at the 2′, 3′, and 4′ positions, with dihydroxy substitution being particularly prevalent. A number of compounds feature an (E)-benzylidene unsaturated side chain, forming conjugated chromone derivatives. In addition, compounds **5**, **6**, **15** and **37** are widely present in the genus *Polygonatum* species, such as *P. odoratum*, *P. kingianum*, *P. cyrtonema*, *P. hunanense*, and *P. verticillatum* [16,17,18,19,20,21,22].

Flavones (**55**–**61**) are characterized by a typical 2-phenylchromen-4-one backbone with a C-2–C-3 double bond. This class mainly consists of C-glycosides and O-glycosides at C-7 or C-8, such as luteolin-7-O-rutinoside (**55**), which has also been isolated from the fruits of *Rosa davurica* [23]. Polyhydroxylated flavones, such as myricetin (**59**), and methylated aglycones, represented by chrysoeriol (**60**), are also identified in this genus and mainly isolated from *P. sibiricum* and *P. cyrtonema* [23,24].

Isoflavones (**62**–**67**) feature the B-ring attached at C-3 instead of C-2, representing a relatively small but distinct group, which are mainly enriched in *P. odoratum* [25,26,27]. Most of them are polyhydroxylated derivatives with methoxyl modifications on the A- and/or B-rings. Tectoridin (**62**) is a typical O-glycosylated isoflavone, while aglycones such as 5,7,4′-trihydroxyisoflavone (**64**) and its methylated analogs demonstrate moderate structural diversification [27].

Chalcones (**68**–**71**) are open-chain flavonoids with a chalcone backbone (1,3-diphenyl-2-propen-1-one). Isoliquiritigenin (**70**) and its glucoside neoisoliquiritin (**69**) are obtained from *P. kingianum* [28,29]. Other constituents include helichrysetin (**68**) and polygonatone D (**71**), which are isolated from the rhizomes of *P. odoratum* and *P. cyrtonema* [21,30].

Flavanones (**72**–**80**) possess a flavan skeleton without the C2-C3 double bond, resulting in a chiral center at C-2. Several methylated and methoxylated derivatives, such as (S)-4′,5,7-trihydroxy-8-methylflavanone (**72**) and farrerol (**73**), have been isolated from *P. cyrtonema* [21].

Isoflavanones (**81**–**84**) feature an isoflavonoid-type skeleton with a saturated C-ring. Most are hydroxylated and methoxylated at the A- and B-rings, and some exist as glucosides, such as 2′,7-Dihydroxy-3′,4′-dimethoxyisoflavan glucoside (**81**), which is isolated from *P. kingianum* [28]. Isoflavanones have been rarely isolated and purified from the *Polygonatum* genus, whereas they are widely present in the Fabaceae family [31].

Flavonols (**85**–**92**) are characterized by a hydroxyl group at C-3 of the flavone backbone, with quercetin and kaempferol as the core aglycones. This class is dominated by O-glycosides. Glycosylation commonly involves glucose, rhamnose, and glucuronic acid, forming di- or trisaccharides, such as rutin (**91**). These compounds are widely distributed in *Polygonatum* species, including *P. sibiricum*, *P. verticillatum*, *P. cyrtonema*, and *P. odoratum* [23,24,27,32,33,34].

Pterocarpan (**93**) is represented by a single compound with a tetracyclic pterocarpan skeleton, showing a unique 6aR,11aR stereochemistry. This compound exhibits a unique structure and rare natural distribution and is exclusively isolated from *P. kingianum* of the genus *Polygonatum*; nevertheless, it has also been identified from *Astragalus membranaceus* and *A. mongholicus* of the Fabaceae family [29], which may serve as a chemotaxonomically significant marker.

**Table 1:** Flavonoid monomers isolated from *Polygonatum*.

NO.	Compounds	Structure	Formula	Source	Ref.
Homoisoflavanones					
1	(3R)-5,7-dihydroxy-3-(4′-hydroxybenzyl)-chroman-4-one	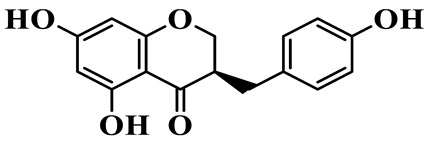	C_16_H_14_O_5_	*P. odoratum*	[16]
2	(3R)-5,7-dihydroxy-8-methyl-3-(2′,4′-dihydroxybenzyl)-chroman-4-one	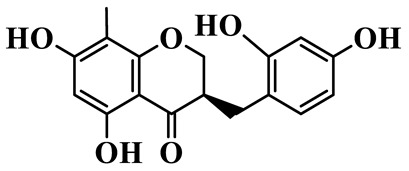	C_16_H_16_O_6_	*P. odoratum*	[16]
3	(3R)-5,7-dihydroxy-3-(2′,4′-dihydroxybenzyl)-chroman-4-one	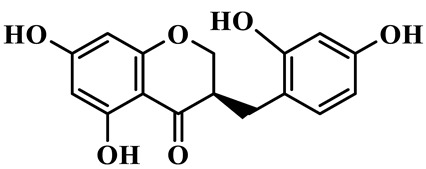	C_15_H_14_O_6_	*P. odoratum*	[16]
4	(3R)-5,7-dihydroxy-8-methyl-3-(4′-hydroxybenzyl)-chroman-4-one	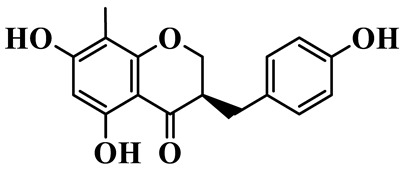	C_16_H_16_O_5_	*P. odoratum*	[16]
5	4′,5,7-Trihydroxy-6,8-dimethylhomoisoflavanone	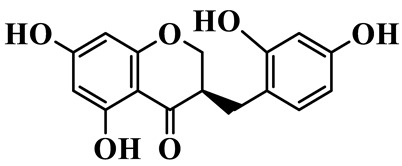	C_18_H_18_O_5_	*P. sibiricum*, *P. hunanense*, *P. cyrtonema*	[17,18,19]
6	Disporopsin	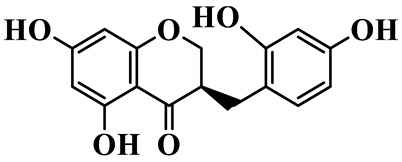	C_16_H_14_O_6_	*P. odoratum*, *P. kingianum*, *P. cyrtonema*	[16,20,21]
7	(3R)-5,7,3′-trihydroxy-4′-methoxy-8-methyl-homoisoflavanone	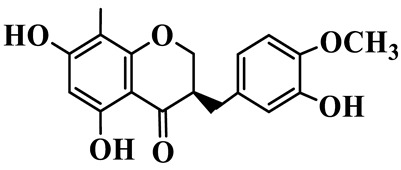	C_18_H_18_O_6_	*P. cyrtonema*	[21]
8	(3R)-5,7,4′-trihydroxy-8-methyl-homoisoflavanone	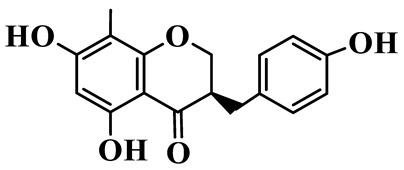	C_17_H_16_O_5_	*P. cyrtonema*	[21]
9	5,7,2′,4′-Tetrahydroxy-6-methyl-homoisoflavanone	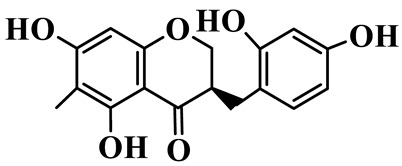	C_17_H_16_O_6_	*P. cyrtonema*	[21]
10	5,7,2′,4′-Tetrahydroxy-6,8-dimethyl-homoisoflavanone	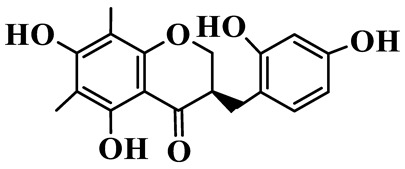	C_19_H_20_O_6_	*P. cyrtonema*	[21]
11	5,7,2′-Trihydroxy-4′-methoxy-6,8-dimethyl-homoisoflavanone	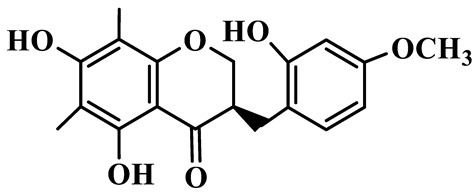	C_20_H_22_O_6_	*P. cyrtonema*	[21]
12	5,7,3′,4′-Tetrahydroxy-6,8-dimethyl-homoisoflavanone	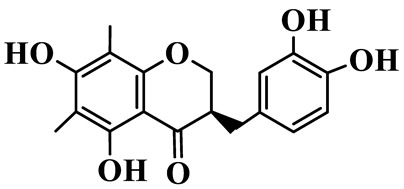	C_18_H_18_O_6_	*P. cyrtonema*	[21]
13	(3R)-5,7,2′,4′-tetrahydroxy-8-methyl-homoisoflavanone	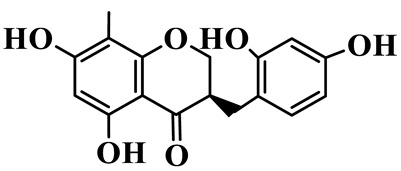	C_18_H_18_O_6_	*P. cyrtonema*	[21]
14	(3R)-5,7-dihydroxy-8-methyl-3-(4′-methoxybenzyl)-chroman-4-one	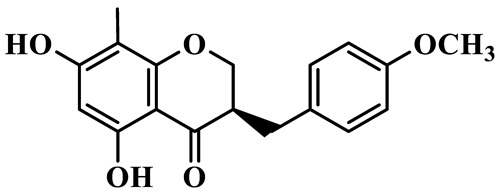	C_18_H_18_O_5_	*P. verticillatum*	[22]
15	(3R)-5,7-dihydroxy-3-(2′-hydroxy-4′-methoxybenzyl)-chroman-4-one	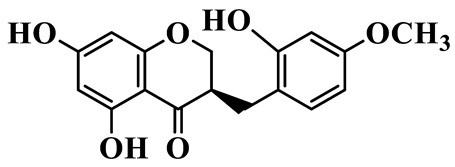	C_17_H_16_O_6_	*P. odoratum*, *P. hunanense*, *P. verticillatum*	[16,18,22]
16	(±)-5,7-Dihydroxy-6,8-dimethyl-3-(2′-hydroxy-4′-methoxybenzyl)-chroman-4-one	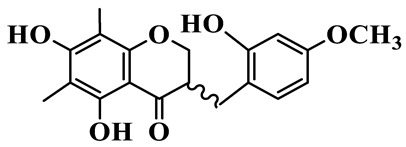	C_19_H_20_O_6_	*P. odoratum*, *P. hunanense*	[18,25]
17	Methylophiopogonanone B	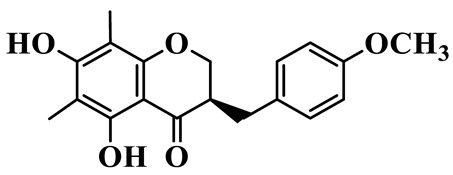	C_19_H_20_O_5_	*P. odoratum*	[25]
18	5,7-Dihydroxy-6,8-dimethyl-3-(3′-hydroxy-4′-methoxybenzyl)-chroman-4-one	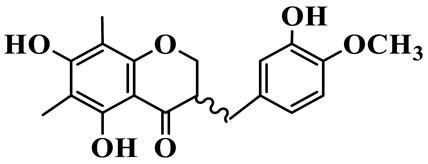	C_19_H_20_O_6_	*P. odoratum*	[25]
19	Ophiopogonanone E	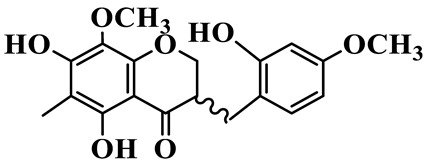	C_19_H_20_O_7_	*P. odoratum*	[25]
20	(E)-7-O-*β*-D-glucopyranoside-5-hydroxy-3-(4′-hydroxybenzylidene)-chroman-4-one	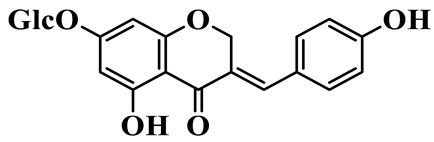	C_22_H_24_O_10_	*P. odoratum*	[25]
21	3R-methylophiopogonanone	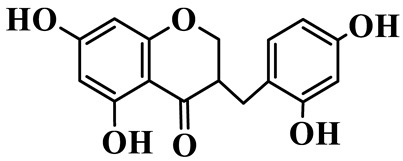	C_19_H_18_O_6_	*P. prattii*	[26]
22	4′-Demethylleucomin7-O-*β*-D-glucopyranoside	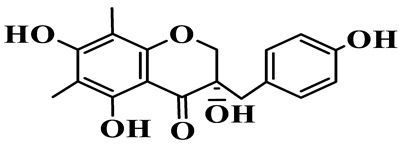	C_22_H_22_O_10_	*P. prattii*	[26]
23	5,7-Dihydroxy-3-(2′,4′-dihydroxybenzyl)-chroma-4-one	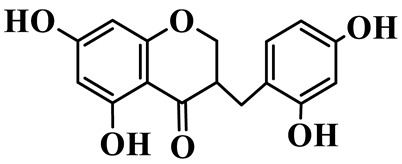	C_16_H_14_O_6_	*P. odoratum*	[27]
24	(3S)-3,5,7-trihydroxy-6,8-dimethyl-3-(4′-hydroxybenzyl)-chroma-4-one	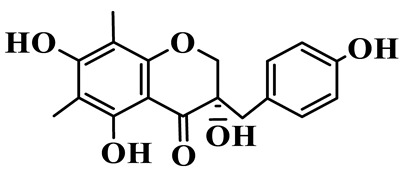	C_18_H_18_O_6_	*P. odoratum*	[27]
25	(3R)-5,7-dihydroxyl-6-methyl-8-methoxyl-3-(4′-hydroxylbenzyl)-chroman-4-one	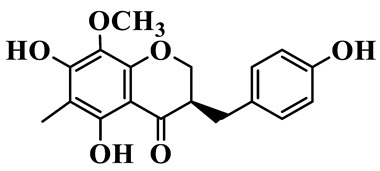	C_18_H_18_O_6_	*P. odoratum*	[35]
26	(3R)-5,7-dihydroxyl-6,8-dimethyl-3-(4′-hydroxylbenzyl)-chroman-4-one	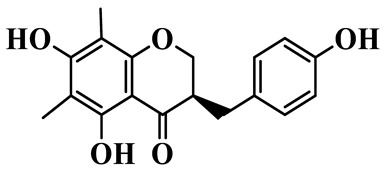	C_18_H_18_O_5_	*P. odoratum*	[35]
27	(3R)-5,7-dihydroxy-6-methoxyl-8-methyl-3-(2′,4′-dihydroxybenzyl)-chroman-4-one	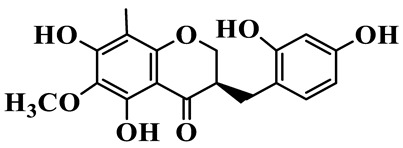	C_18_H_18_O_7_	*P. odoratum*	[36]
28	5,7-Dihydroxy-6-methyl-3-(2′,4′-dihydroxybenzyl)-chroman-4-one	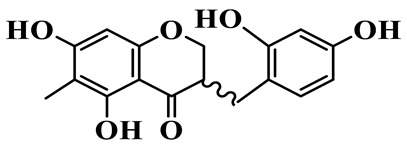	C_17_H_16_O_6_	*P. odoratum*	[36]
29	5,7-Dihydroxy-6-methoxyl-8-methyl-3-(2′,4′-dihydroxybenzyl)-chroman-4-one	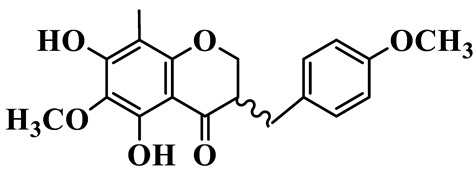	C_19_H_20_O_6_	*P. odoratum*	[36]
30	(3R)-5,7-dihydroxyl-6-methyl-3-(4′-hydroxylbenzyl)-chroman-4-one	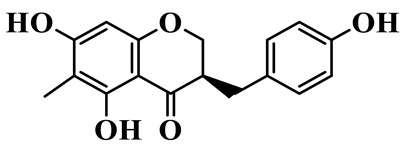	C_17_H_16_O_5_	*P. odoratum*, *P. kingianum*	[37,38]
31	(±)-5,7-Dihydroxy-6,8-dimethyl-3-(2′,4′-dihydroxybenzyl)-chroman-4-one	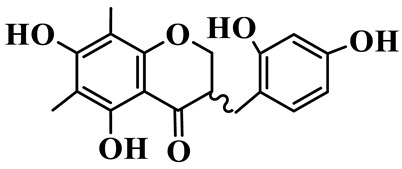	C_18_H_18_O_6_	*P. kingianum*	[38]
32	(3R)-5,7-dihydroxy-8-methyl-3-(2′-hydroxy-4′-methoxybenzyl)-chroman-4-one	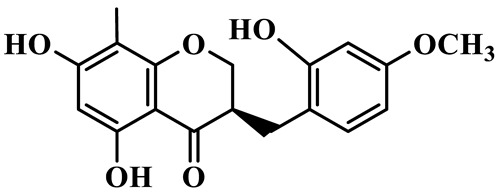	C_18_H_18_O_6_	*P. sibiricum*	[38]
33	Ophiopogonanone G	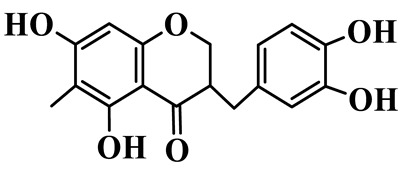	C_17_H_16_O_6_	*P. hunanense*, *P. kingianum*	[18,38]
34	Polygonatone A	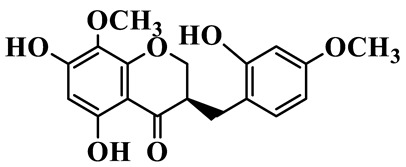	C_18_H_18_O_7_	*P. odoratum*	[39]
35	Polygonatone B	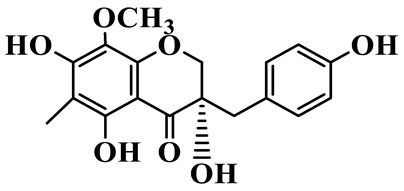	C_18_H_18_O_7_	*P. odoratum*	[39]
36	Polygonatone C	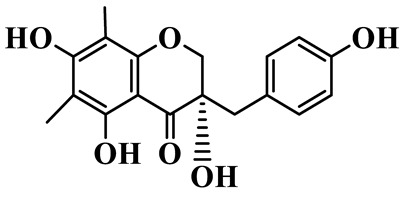	C_18_H_18_O_6_	*P. odoratum*	[39]
37	(3R)-5,7-dihydroxy-8-methyl-3-(2′-hydroxy-4′-methoxybenzyl)-chroman-4-one	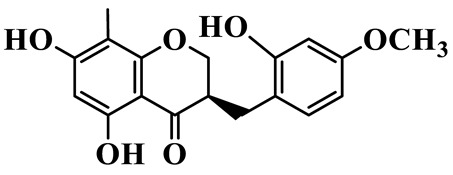	C_18_H_18_O_6_	*P. odoratum*, *P. kingianum*, *P. verticillatum*	[22,38,40]
38	Odoratumone A	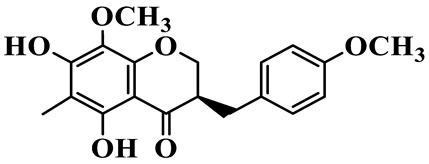	C_19_H_20_O_6_	*P. odoratum*	[41]
39	Odoratumone B	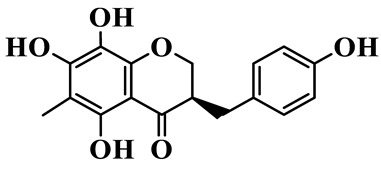	C_17_H_16_O_6_	*P. odoratum*	[41]
40	(E)-3-(3′,4′-dihydroxybenzylidene)-5,7-dihydroxy-6,8-dimethylchroman-4-one	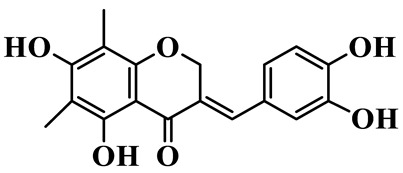	C_17_H_16_O_6_	*P. odoratum*	[42]
41	(E)-3-(3′,4′-dihydroxybenzylidene)-5,7-dihydroxy-8-methoxy-6-methylchroman-4-one	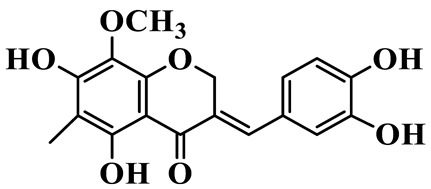	C_17_H_16_O_7_	*P. odoratum*	[42]
42	(3S)-3,5,7-trihydroxy-6-methyl-3-(4′-methoxybenzyl)-chroman-4-one	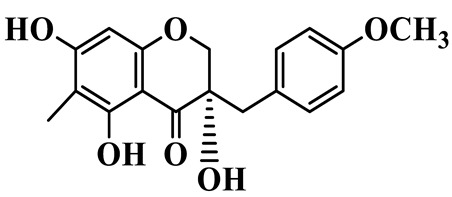	C_18_H_18_O_6_	*P. odoratum*	[43]
43	(E)-5,7-dihydroxy-6,8-dimethyl-3-(4′-hydroxybenzylidene)-chroman-4-one	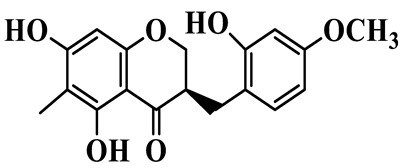	C_18_H_16_O_5_	*P. odoratum*, *P. cyrtonema*	[25,44]
44	Polygonatone H	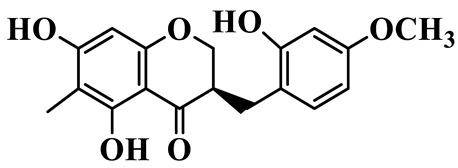	C_18_H_18_O_6_	*P. cyrtonema*, *P. hunanense*	[18,44]
45	(3R)-5-hydroxy-7-methoxy 3-(3′,4′-dihydroxybenzyl)- chroman-4-one	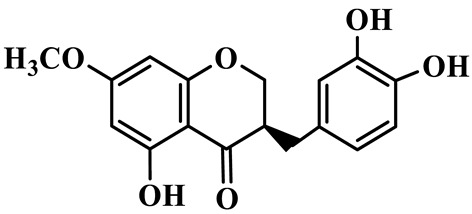	C_17_H_16_O_6_	*P. sibiricum*	[45]
46	Polygonatone I	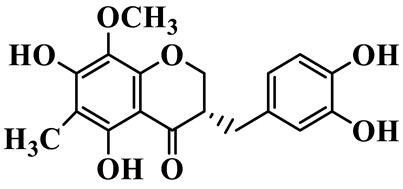	C_18_H_18_O_7_	*P. sibiricum*	[45]
47	Polygonatone J	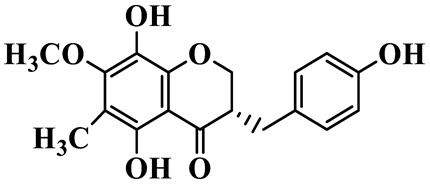	C_18_H_18_O_6_	*P. sibiricum*	[45]
48	Polygonatone K	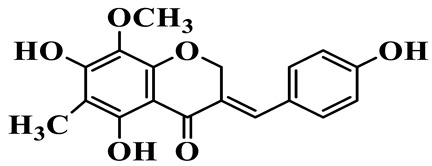	C_18_H_16_O_6_	*P. sibiricum*	[45]
49	Polygonatone L	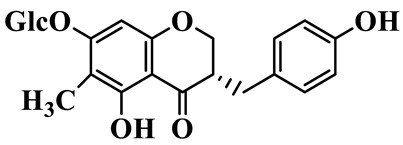	C_23_H_28_O_10_	*P. sibiricum*	[45]
50	Polygonatone M	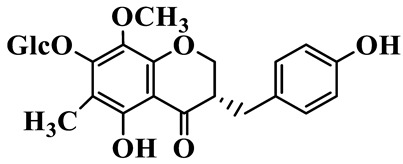	C_24_H_28_O_11_	*P. sibiricum*	[45]
51	Polygonatone N	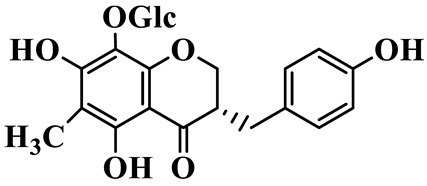	C_23_H_26_O_11_	*P. sibiricum*	[45]
52	4′-Demethyleucomin	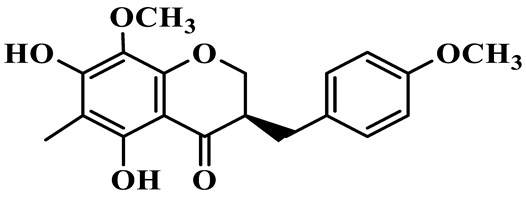	C_16_H_12_O_5_	*P. rhizoma*	[46]
53	(3R)-brevifolin	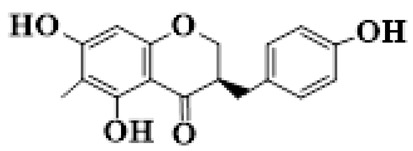	C_17_H_16_O_5_	*P. rhizoma*	[46]
54	(3R)-5,7-dihydroxyl-6-methyl-8-methoxyl-3-(4′-methoxybenzyl)-chroman-4-one	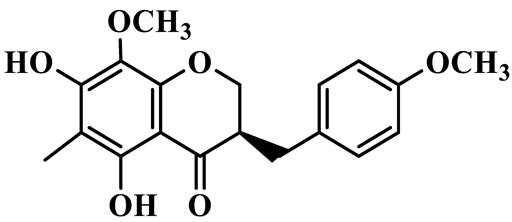	C_19_H_20_O_5_	*P. odoratum*	[47]
Flavones					
55	Luteolin-7-O-rutinoside	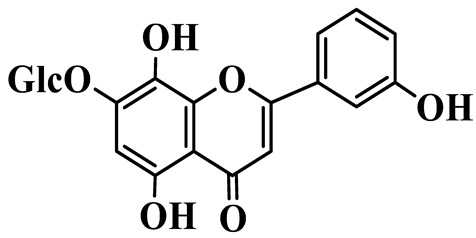	C_27_H_30_O_5_	*P. cyrtonema*	[23]
56	Isovitexin	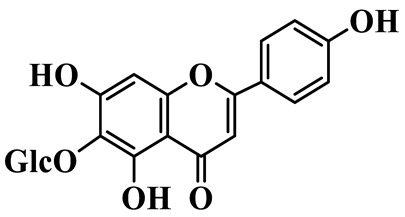	C_21_H_20_O_10_	*P. sibiricum*	[24]
57	Isovitexin 8-C-*β*-D-glucoside	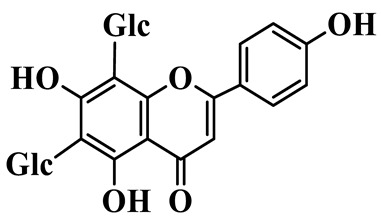	C_27_H_30_O_15_	*P. sibiricum*	[24]
58	Apigenin-7-O-*β*-D-glucopyranoside	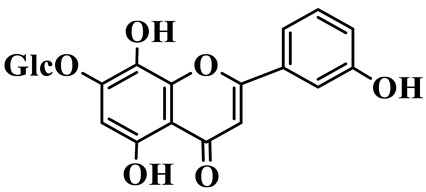	C_21_H_20_O_10_	*P. sibiricum* *P. cyrtonema*	[23,32]
59	Myricetin	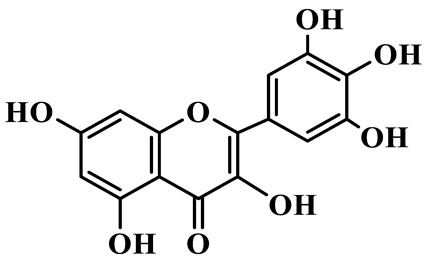	C_15_H_10_O_8_	*P. sibiricum*	[32]
60	Chrysoeriol	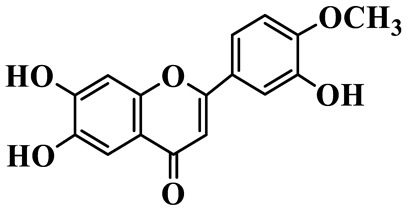	C_16_H_12_O_6_	*P. odoratum*, *P. sibiricum*	[24,36]
61	Apigenin-8-C-glucoside	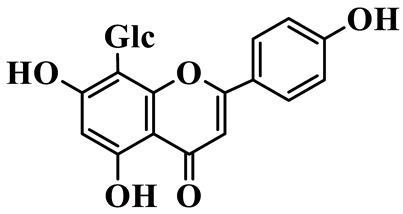	C_21_H_20_O_10_	*P. sibiricum*	[48]
Isoflavones					
62	Tectoridin	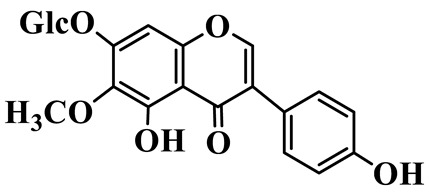	C_22_H_22_O_11_	*P. odoratum*	[25]
63	2′,5-Dihydroxy-7-hydroxymethyl isoflavone	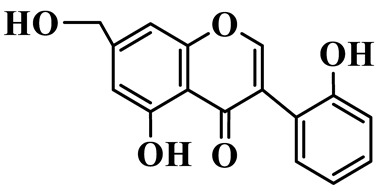	C_16_H_12_O_5_	*P. prattii*	[26]
64	5,7,4′-Trihydroxy isoflavone	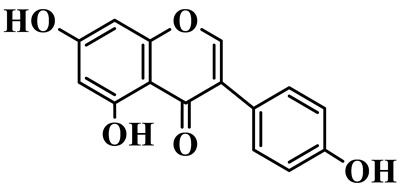	C_15_H_10_O_5_	*P. odoratum*	[27]
65	5,7,4′-Trihydroxy-6-methoxy isoflavone	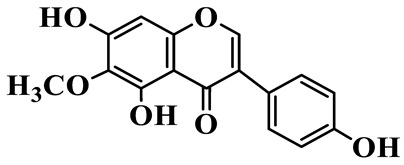	C_16_H_12_O_6_	*P. odoratum*	[27]
66	5,7,4′-Trihydroxy-6,3′-dimethoxy isoflavone	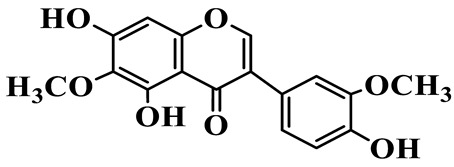	C_17_H_14_O_7_	*P. odoratum*	[27]
67	4′,7-Dihydroxy-3′-methoxyisoflavone	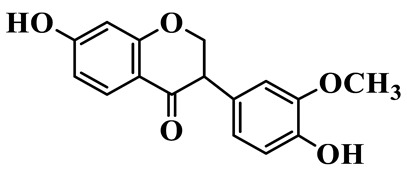	C_16_H_14_O_5_	*P. kingianum*	[29]
Chalcones					
68	Helichrysetin	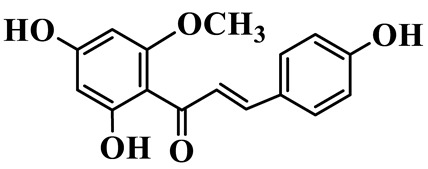	C_16_H_14_O_5_	*P. cyrtonema*	[21]
69	Neoisoliquiritin	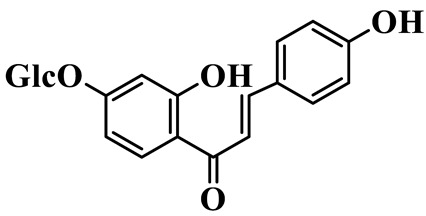	C_20_H_22_O_9_	*P. kingianum*	[28]
70	Isoliquiritigenin	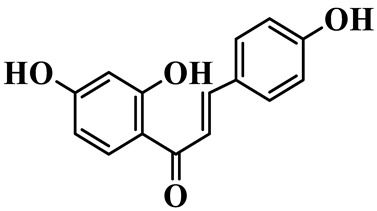	C_15_H_12_O_4_	*P. kingianum*	[29]
71	Polygonatone D	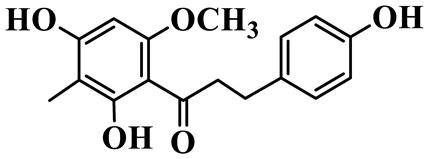	C_17_H_18_O_5_	*P. odoratum*	[30]
Flavanones					
72	(S)-4′,5,7-trihydroxy-8-methyl-flavanone	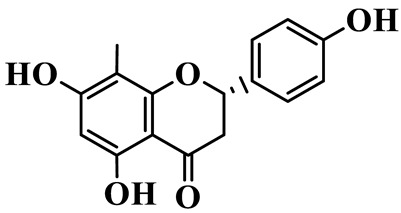	C_16_H_14_O_5_	*P. cyrtonema*	[21]
73	Farrerol	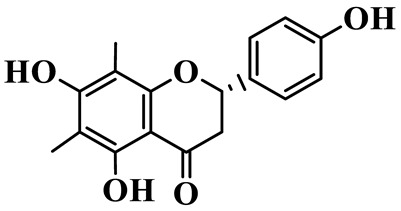	C_17_H_16_O_5_	*P. cyrtonema*	[21]
74	5,7-Dihydroxy-8-methyl-4′-methoxyflavanone	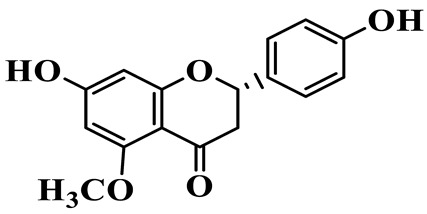	C_17_H_16_O_5_	*P. cyrtonema*	[21]
75	Hesperidin	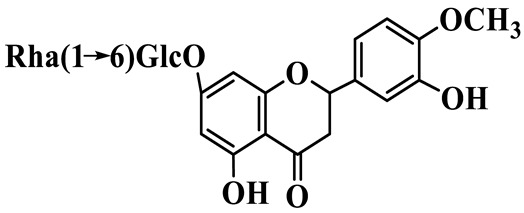	C_28_H_34_O_15_	*P. odoratum*	[25]
76	7,4′-Dihydroxy-5-methoxy flavanones	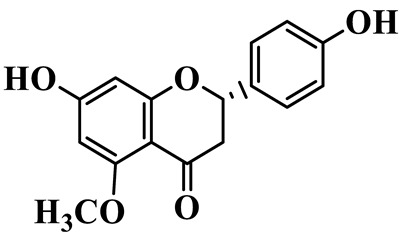	C_15_H_14_O_5_	*P. prattii*	[26]
77	Neoliquiritin	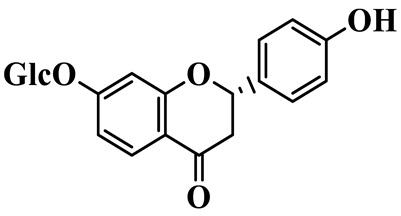	C_20_H_20_O_9_	*P. kingianum*	[28]
78	Liquiritin	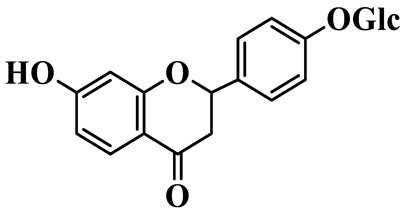	C_15_H_12_O_4_	*P. kingianum*	[29]
79	Naringenin	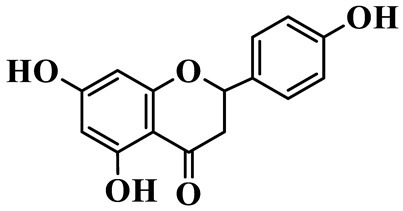	C_15_H_12_O_5_	*P. rhizome*, *P. cyrtonema*	[21,47]
80	Liquiritigenin	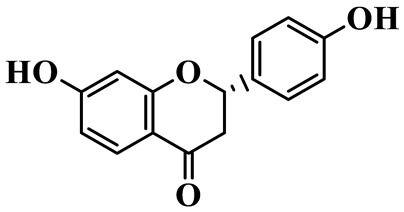	C_15_H_12_O_4_	*P. alte-lobatum*, *P. kingianum*, *P. odoratum*	[28,29,49]
Isoflavanones					
81	2′,7-Dihydroxy-3′,4′-dimethoxyisoflavan glucoside	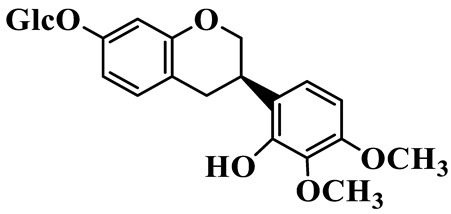	C_23_H_28_O_10_	*P. kingianum*	[28]
82	2′,7-Dihydroxy-3′,4′-dimethoxyisoflavan	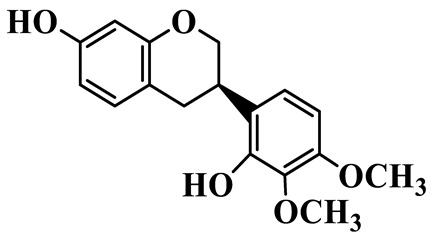	C_17_H_18_O_5_	*P. kingianum*	[28]
83	5,4′-Dihydroxy-7-methoxy-6-methylflavane	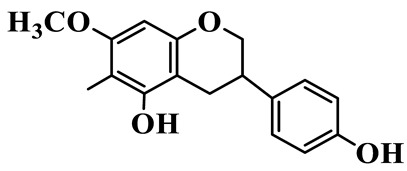	C_17_H_18_O_4_	*P. odoratum*	[36]
84	Isomucronulatol	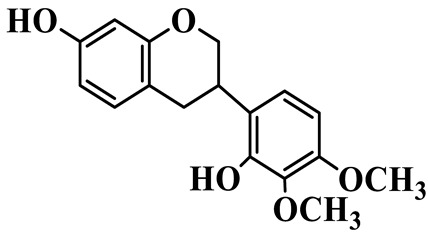	C_17_H_18_O_5_	*P. kingianum*	[50]
Flavonols					
85	Quercetin 3-O-*β*-D-glucuronide	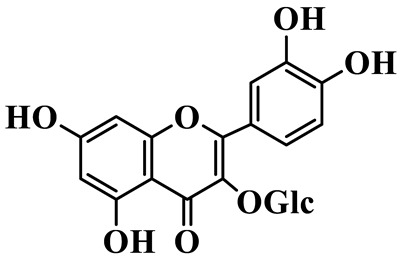	C_21_H_20_O_12_	*P. cyrtonema*	[23]
86	Kaempferol-7-O-*β*-D-glucopyranoside	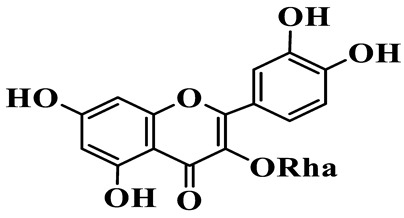	C_21_H_20_O_11_	*P. cyrtonema*	[23]
87	Quercetin 3-O-*α*-L-rhamnoside	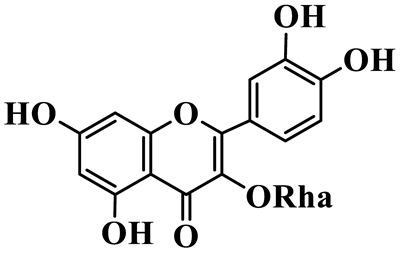	C_21_H_20_O_11_	*P. sibiricum*	[24]
88	Isorhamnetin-3-O-(6″-O-*α*-L-rhamnopyransoyl)-*β*-D-glucopyranoside	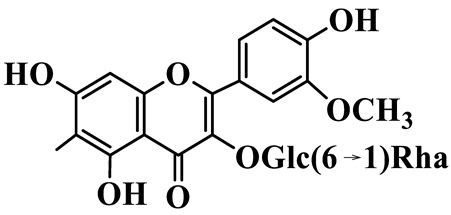	C_29_H_32_O_16_	*P. odoratum*	[27]
89	Isoquercetin	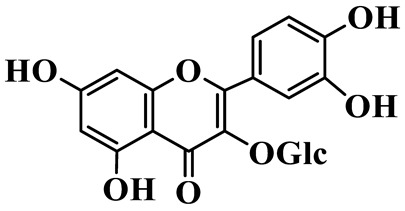	C_21_H_20_O_12_	*P. sibiricum*,	[33]
90	Hyperoside	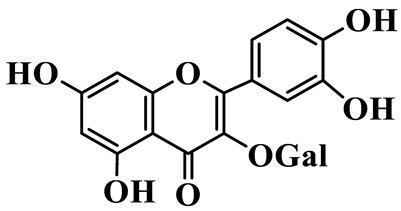	C_21_H_20_O_12_	*P. sibiricum*	[33]
91	Rutin	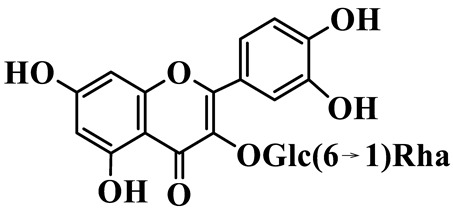	C_27_H_30_O_16_	*P. sibiricum*, *P. verticillatum*, *P. cyrtonema*	[33,34,43]
92	Kaempferol	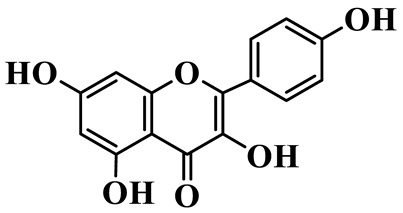	C_15_H_10_O_6_	*P. sibiricum*, *P. verticillatum*	[32,34]
Pterocarpan					
93	(6aR,11aR)-3,9-dimethoxy-10-hydroxypterocarpan	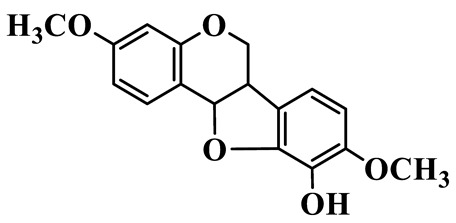	C_17_H_16_O_5_	*P. kingianum*	[29]

Note: Glc: *β*-D-glucopyranosyl; Xyl: xylopyranoside; Gal: Galactopyranose; Rha: *α*-L-rhamnopyranose; GlcA: *β*-D-Glucopyranuronic acid.

## 4. Biosynthesis of Flavonoids

Flavonoids are a major class of plant secondary metabolites with diverse biological functions, and their biosynthetic pathway is highly conserved across plant species, starting from the aromatic amino acid phenylalanine [51,52]. As illustrated in the pathway, the process begins with the phenylpropanoid pathway, which converts phenylalanine into the key intermediate *p*-coumaroyl-CoA, which then enters the flavonoid-specific biosynthetic branch that generates various flavonoid subclasses [53]. The initial step is catalyzed by phenylalanine ammonia-lyase (PAL), which deaminates phenylalanine to form cinnamic acid. Subsequently, cinnamate 4-hydroxylase (C4H) introduces a hydroxyl group at the C-4 position of cinnamic acid, producing *p*-coumaric acid. Then, 4-coumarate CoA ligase (4CL) activates *p*-coumaric acid by linking it to coenzyme A, yielding *p*-coumaroyl-CoA, which is the core precursor for flavonoid biosynthesis [54]. From this point, the pathway diverges into flavonoid-specific reactions. Chalcone synthase (CHS) condenses one molecule of *p*-coumaroyl-CoA with three molecules of malonyl-CoA, forming naringenin chalcone, the first flavonoid intermediate. Chalcone isomerase (CHI) then stereospecifically converts the open-chain chalcone into the cyclic flavanone naringenin, which serves as the central branching point for downstream flavonoid subclasses.

**Figure 1:**
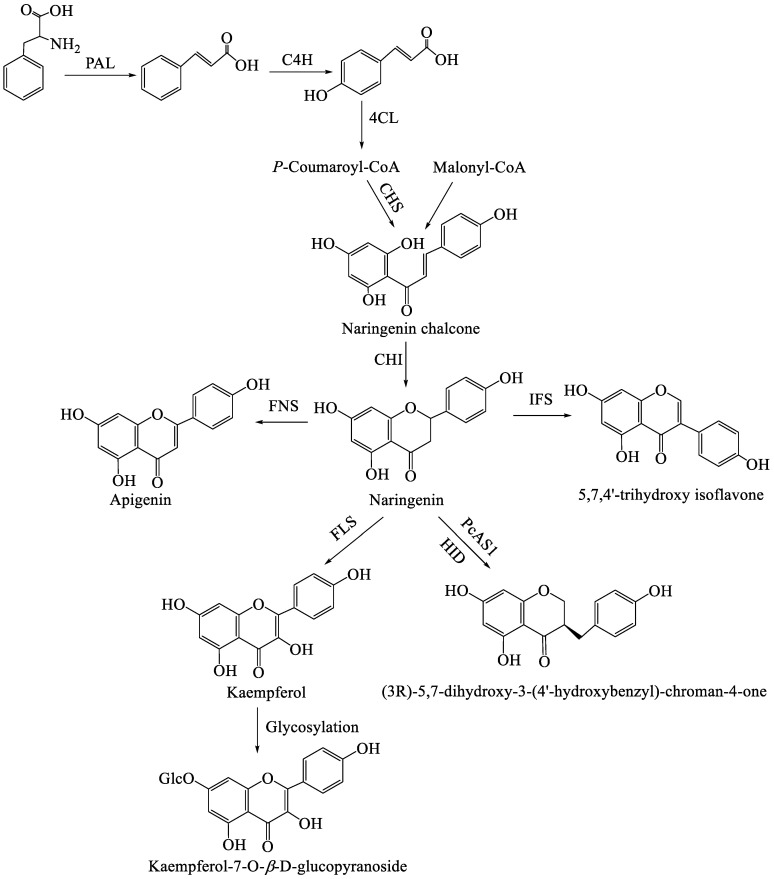
The putative biosynthetic pathway of flavonoids in *Polygonatum* plants. PAL, phenylalanine ammonialyase; C4H, cinnamate 4-hydroxylase; 4CL, 4-coumarate-CoA ligase; CHS, chalcone synthase; CHI, chalcone isomerase; FNS, flavone synthase; IFS, isoflavone synthase; FLS, flavonol synthase; PcAS1, Polygonatum cyrtonema cyclase 1; HID, 2-hydroxyisoflavanone dehydratase.

Naringenin is transformed into distinct flavonoid types through the action of various enzymes [55,56]: Flavone synthase (FNS) oxidizes naringenin to form apigenin, a representative flavone. Isoflavone synthase (IFS) catalyzes a 1,2-aryl migration reaction, converting naringenin into 5,7,4′-trihydroxy isoflavone, an isoflavonoid derivative. Flavonol synthase (FLS) introduces a double bond and a hydroxyl group at the C-3 position, generating kaempferol, a common flavonol. In addition, the specific enzyme *Polygonatum cyrtonema* cyclase 1 (PcAS1) in *Polygonatum* can catalyze the 3-benzylation migration of naringenin to generate the 3-benzyl-4-chromanone mother nucleus unique to homoisoflavonoids [57]. Finally, flavonols such as kaempferol undergo further modifications such as glycosylation, where a glucose moiety is attached to the hydroxyl group at the C-7 position, resulting in kaempferol-7-O-*β*-D-glucopyranoside [58]. This glycosylation step may enhance the stability and solubility of flavonoids, facilitating their storage and transport in plant tissues. The possible biosynthetic pathways of flavonoids in the genus *Polygonatum* are shown in Figure 1.

## 5. Pharmacological Effects

### 5.1. Antioxidant Activity

Flavonoids, a large class of polyphenolic secondary metabolites widely distributed in plants, exhibit remarkable antioxidant properties and have become a research hotspot in the fields of nutrition, medicine, and food science [59] (for in vitro experiments, see Table 2). Zhu et al. [60] reported that the total flavonoids from *P. odoratum* (TFPo) exhibited strong DPPH radical scavenging capacity, and this activity was significantly enhanced after the interaction between TFPo and iron salts. Another study also revealed that the total flavonoids from *P. sibiricum* (TFPs) possessed potent scavenging effects on DPPH and ABTS radicals, with corresponding 50% inhibitory concentration (IC_50_) values of 27.55 and 11.47 μg/mL. Additionally, TFPs exhibited strong ferrous ion-chelating activity, with an IC_50_ value of 32.26 μg/mL [61]. Horng et al. [62] evaluated the antioxidant activity of *P. alte-lobatum* using a DPPH assay. The results showed that the ethanolic extract of *P. alte-lobatum* exhibited dose-dependent free radical scavenging effects on DPPH, with an IC_50_ value of 9 μg/mL; additionally, total flavonoids were one of its main active ingredients, which suggests that the flavonoid components in the *Polygonatum* genus make a significant contribution to its antioxidant activity. Meanwhile, another study demonstrated that the DPPH and ABTS free radical scavenging activities of TFPs exhibited a positive concentration-dependent relationship. At a concentration of 0.5 mg/mL, the scavenging effects reached their peak, with values of 84.3% and 81.5% for DPPH and ABTS radicals, respectively [63]. Eleven antioxidant homoisoflavanones were isolated from *P. odoratum* via high-speed counter-current chromatography (HSCCC), with all isolated homoisoflavonoids displaying potent antioxidant activities, including compounds **2**, **3**, and **27**, which possess dihydroxylated B-rings and showed stronger antioxidant effects (IC_50_ = 3.8 ± 0.5, 4.9 ± 0.3, and 3.9 ± 0.4 μg/mL, respectively) than ascorbic acid (IC_50_ = 5.3 ± 0.6 μg/mL) [16]. The extracts of *P. odoratum* were evaluated using three complementary assays. Among all extracts tested, the crude flavonoid extract (FE) displayed the most potent antioxidant activity, with corresponding IC_50_ values of 0.06 ± 0.035 mg/mL for DPPH radical scavenging and 0.68 ± 0.030 mg/mL for hydroxyl radical scavenging. Subsequently, two C-methylated homoisoflavanones (compounds **25** and **30**) were purified from FE. The bioactivity evaluation revealed that both compounds exhibited remarkable DPPH radical scavenging and reducing power activities; however, they showed no obvious hydroxyl radical scavenging capacity. Compound **25** (IC_50_ = 5.90 ± 0.150 μg/mL) exhibited a nearly twofold higher DPPH radical scavenging activity than compound **6** (IC_50_ = 11.64 ± 0.296 μg/mL), and its activity was comparable to that of rutin (IC_50_ = 5.79 ± 0.140 μg/mL) [64]. The total flavonoids from wine-processed *Polygonatum* showed scavenging effects on DPPH, OH, and ABTS free radicals, with IC_50_ values of (19.43 ± 0.96) μg/mL, (4.46 ± 0.14) μg/mL, and (31.57 ± 0.14) μg/mL, respectively [65]. Other studies have also confirmed that the scavenging capacity of TFPs against DPPH and ABTS free radicals exhibited an increasing trend within a certain concentration range, showing a clear dose-dependent effect. At mass concentrations of 2 and 3 mg/mL, the ABTS radical scavenging ability of TFPs was comparable to that of vitamin C [66]. Compounds **82**, **84**, and **87** were isolated from *P. sibiricum*, which displayed strong antioxidant activities, as evaluated by DPPH, superoxide and ABTS assays. In the FRAP assay, compound **84** exhibited the most potent antioxidant capacity (3898.88 ± 23.23 mM TE/g) [33]. Wei et al. [67] reported that TFPs showed strong DPPH free radical scavenging activity in the concentration range of 0.1–0.5 mg/mL and exerted the scavenging effect in a concentration-dependent manner. At 0.5 mg/mL, the DPPH scavenging rate of TFPs was 91%. Another study showed that the total flavonoids from *P. kingianum* (TFPk) exhibited strong scavenging activities against DPPH and ABTS free radicals, with EC_50_ values of 2.11 and 1.63 mg/mL; the results demonstrated that TFPk possessed significant antioxidant effects and could be used as a potential raw material in the food industry [68]. Subsequently, Wang et al. [69] investigated the purification process and antioxidant activity of TFPk. The results showed that after purification by macroporous resin AB-8, the scavenging rates of TFPk against DPPH and ABTS free radicals reached 82.44% and 86.22%, respectively, which were significantly higher than those before purification. The hydroxyl configuration on the B-ring is the key determinant in free radical scavenging by donating hydrogen atoms and electrons to hydroxyl, peroxyl, and peroxynitrite radicals.

### 5.2. Anti-Diabetic Activity

As characteristic bioactive constituents of medicinal plants, flavonoids can safely modulate blood glucose levels [70]. In in vitro experiments, TFPk extracted by Wang et al. [68] exhibited EC_50_ values of 1.70 and 2.69 mg/mL in inhibiting *α*-glucosidase and *α*-amylase, respectively, indicating its promising hypoglycemic activity. Adenosine monophosphate (AMP)–AMPK serves as a key cellular energy sensor and a central regulator of metabolic homeostasis, making it a core target in research on diabetes and associated metabolic disorders [71]. The effects of homoisoflavonoids on adenosine monophosphate-activated kinase (AMPK) activation were subsequently investigated. In rat liver epithelial IAR-20 cells, treatment with these flavonoids elevated the levels of phosphorylated AMPK and acetyl-CoA carboxylase, both of which represent active forms of the respective proteins. Glucose uptake was notably enhanced by compounds **25**, **26**, **30**, and **71**, partially through triggering the AMPK signaling pathway [72]. Another study also demonstrated that homoisoflavonoids (**25**, **26**, and **30**) derived from *P. odoratum* rhizomes act as potent inhibitors of glucose transporter 2 (GLUT2); they reduced blood glucose levels by suppressing intestinal glucose transport pathways and inhibiting sodium-dependent glucose uptake. Consequently, they can be incorporated into functional foods or beverages to effectively modulate postprandial blood glucose concentrations [73]. Zhang et al. [25] isolated flavonoid glycosides from the rhizomes of P. odoratum. Glucose uptake assays in 3T3-L1 mouse embryonic fibroblast cells (3T3-L1) adipocytes showed that all flavonoid glycosides exerted insulin-sensitizing effects, indicating that flavonoids may have potential as insulin sensitizers. Further studies found that isorhamnetin could significantly increase glucose consumption in insulin-resistant (IR) HepG2 cell models, upregulate the protein expression levels of PI3K and AKT1, and downregulate the protein expression levels of VEGF and mTOR [74]. Compounds **25**, **26**, and **38** were isolated from *P. odoratum* rhizomes. Their inhibitory effects on the formation of advanced glycation end products (AGEs) were investigated using in vitro bioassays, exhibiting stronger inhibitory activity against AGE formation than the positive control aminoguanidine [48]. Peroxisome proliferator-activated receptor gamma (PPARγ) belongs to the nuclear receptor superfamily, and its agonists serve as anti-hyperglycemic agents [75]. Homoisoflavonoids from *P. odoratum* were tested for PPARγ activity using fluorescence polarization competitive binding and transient transfection reporter assays. Compound **25** (IC_50_ = 12 μM) and compound **26** (IC_50_ = 7 μM) showed moderate binding affinity to the PPARγ ligand-binding domain. Both compounds dose-dependently transactivated PPARγ-dependent promoters. Molecular modeling revealed that they bound to the PPARγ ligand-binding pocket similarly to indeglitazar, a known PPARγ agonist [76]. In in vivo experiments, Shu et al. [77] investigated the hypoglycemic activity of TFPo in alloxan-induced diabetic rats. The results demonstrated that TFPo exhibited significant dose-dependent anti-diabetic activity and represents one of the major active constituents responsible for its hypoglycemic effects. Subsequent studies showed the anti-diabetic activity of TFPs via determination of blood glucose (BG) with a one-touch glucometer and insulin levels using a radioimmunoassay kit in alloxan-induced diabetic rats and *α*-amylase inhibitory activity by *α*-amylase inhibition assay in vitro. The results suggest that TFPs effectively improved blood glucose control. Daily administration of TFPs at 50–200 mg/kg body weight for 30-day treatment with the same doses significantly decreased fasting blood glucose in alloxan-induced diabetic rats, and α-amylase inhibition assays in vitro further demonstrated that TFPs markedly inhibited α-amylase activity in a dose-dependent manner [78]. The representative anti-diabetic activity of flavonoids from the genus *Polygonatum* is summarized in Table 2.

### 5.3. Anticancer Activity

Flavonoids are a class of natural polyphenolic active substances found in plants and have shown significant anticancer activity; furthermore, the molecular basis of their antitumor effects is their intervention in key signaling pathways such as PI3K/Akt/mTOR, MAPK/ERK, NF-κB, and p53 [79,80,81,82,83] (For in vitro experiments, see Table 2). Compound **1** increased the proportion of cells in the G2/M phase and induced apoptosis in human lung cancer A549 cells via the p38 mitogen-activated protein kinase (MAPK) pathway and mitochondria-mediated apoptotic signaling, which are closely associated with the antitumor mechanism of homoisoflavone [84]. Furthermore, compound **25** exerted significant dose-dependent inhibitory effects on the proliferation of A549 cells and induced their apoptosis by regulating mitochondria-caspase-dependent pathways and endoplasmic reticulum (ER) stress responses. Compound **25** also triggered G2/M cell cycle arrest through activation of the p38/p53 signaling pathway [85]. In addition, compound **26** induced Bcl-2 phosphorylation in breast tumor cells, caused G2/M cell cycle arrest, upregulated the expression of p21 and p53 proteins and decreased cell viability, demonstrated via a clonogenic assay [35]. Compounds **25**–**28** showed cytotoxicity against K562, A549, and HCT-15 tumor cells in the MTT colorimetric assay, the leukocyte elastase inhibitor screening model, and the dihydroorotate dehydrogenase inhibitor screening model [86]. Another study also showed that homoisoflavone could induce B-cell lymphoma 2 (Bcl-2) phosphorylation, apoptosis, and G2/M cell cycle arrest in breast tumor cells [87]. Xuan et al. [38] isolated four homoisoflavonoids from *P. kingianum*. Among them, compounds **30**, **33**, and **37** exhibited cytotoxic activity against human hepatocellular carcinoma cells HepG2 and human non-small cell lung cancer cells A549 using the CCK-8 assay. Furthermore, compound **37** showed significant inhibitory effects on mouse tumor cells, and its IC_50_ value against mouse macrophages was determined to be 17.99 ± 1.45 μmol/L. In addition, compound **80** isolated from *P. sibiricum* was investigated in breast cancer (BC) cells. The results demonstrated that liquiritigenin downregulated HSP90 and Snail expression, upregulated E-cadherin expression, and inhibited the proliferation, migration, and invasion of BC cells. [88]. Recently, Wang et al. [89] explored the potential mechanism of *P. sibiricum* against hepatocellular carcinoma based on network pharmacology and molecular docking. The results indicated that the main active components of *P. sibiricum* may be baicalein, liquiritin, and higenamine, which inhibit proliferation, induce apoptosis and interfere with the metastasis of hepatocellular carcinoma by regulating PI3K/Akt/mTOR, MAPK, Wnt and other signaling pathways through multiple targets. Meanwhile, in in vivo experiments, homoisoflavanone significantly suppressed tumor growth in a colorectal cancer xenograft mouse model, with no observable systemic toxicity. Collectively, these findings identify homoisoflavanone as a promising plant-derived therapeutic candidate that targets DNA integrity and mitochondrial homeostasis to impede colorectal cancer progression [90]. Wan et al. [21] evaluated isolated flavonoids’ cytotoxicity against five human cancer cell lines (HepG2, HCT-116, AGS, U-87 MG, and PC-12) via the MTT assay, with paclitaxel as the positive control. The results indicate that compound **16** displayed potent and selective cytotoxicity toward AGS and PC-12 cells, with corresponding IC_50_ values of 7.2 and 9.8 μM, respectively. Notably, none of the isolated compounds showed activity against the human hepatocellular carcinoma cell line HepG2. Meanwhile, compound **44** exhibited cytotoxic effects against four tumor cell lines, with IC_50_ values ranging from 2.2 to 9.8 μM. The antitumor effects of flavonoids are closely related to their structural features. Hydroxyl substitution, ring conjugation and substituent types jointly regulate their anticancer capacity.

### 5.4. Anti-Inflammatory Activity

Recent studies have demonstrated that flavonoids can inhibit key regulatory enzymes and transcription factors, which are critical for modulating inflammation-related mediators [91]. Moreover, flavonoids are well-recognized as potent antioxidants capable of alleviating tissue injury and fibrosis. Consistently, accumulating evidence from in vitro and in vivo studies has confirmed that flavonoids effectively suppress the onset and progression of inflammatory disorders [92] (For in vitro experiments, see Table 2). Compounds **89**–**91** exhibited concentration-dependent inhibition of iNOS and NO production, as well as TNF-*α* and IL-6 secretion, verifying their potent anti-inflammatory effects. Molecular docking further demonstrated that compound **91** displayed a significantly higher binding affinity than others and quercetin (positive control), consistent with its strongest anti-NO activity [33]. Liu et al. [93] investigated the effective components and underlying mechanism of *P. cyrtonema* against inflammatory fatigue using LC-MS analysis and network pharmacology. The results revealed that flavonoids such as liquiritigenin were among its main active constituents. Enrichment analysis further revealed that the 5-HT aminergic synapse, calcium signaling, JAK-STAT, and NF-κB pathways constituted the major regulatory mechanisms. Subsequent cell assays confirmed that *P. cyrtonema* significantly suppressed LPS-induced inflammatory cytokine secretion in macrophages, thereby demonstrating its potent anti-inflammatory properties. Niu et al. [94] investigated the effects of high-pressure steam heating at different temperatures on the active components of *P. kingianum*, demonstrating that high-pressure steam processing significantly increased the active ingredient content of *P. kingianum*, as well as its anti-inflammatory and antioxidant activities. Compounds **55**, **85**, **86**, and **91** were isolated from the massive rhizomes of *P. cyrtonema*. Biologically, all compounds were evaluated for their anti-inflammatory activities by inhibiting NO production in LPS-stimulated RAW 264.7 cells in vitro. The results indicated that these flavonoids exhibited moderate inhibitory effects on NO production, with IC_50_ values ranging from 8.28 to 41.85 μmol/L [23]. Homoisoflavonoids’ anti-inflammatory activities were evaluated using a lipopolysaccharide (LPS)-induced mouse macrophage (RAW 264.7) model, showing that these homoisoflavonoids could inhibit NO production, and compound **31** exhibited promising anti-inflammatory activity. At a concentration of 16 μmol/L, the NO inhibition rate of compound **31** reached 91.16% ± 3.51%, which was significantly higher than that of the positive control dexamethasone (64.81% ± 1.71%) [38]. Glycosylation and double-bond conjugation effectively inhibit inflammatory mediators and alleviate oxidative inflammation.

### 5.5. Antibacterial Activity

Flavonoids from the genus *Polygonatum* showed inhibitory activity against a broad spectrum of bacteria [95] (For in vitro experiments, see Table 2). Khan et al. [96] demonstrated that the total flavonoids of *P. verticillatum* (TFPv) exhibited antibacterial effects against *Escherichia coli*, *Salmonella typhi*, *Shigella flexneri*, and *Staphylococcus aureus*, with minimum inhibitory concentrations (MICs) of 1.5–40, 3–6, 3–40, and 75–80 μg/mL, respectively. Subsequently, they further investigated the antibacterial and antifungal activities of the crude methanol extract from the aerial parts of *P. verticillatum* and its various solvent fractions. Phytochemical screening confirmed the presence of high levels of flavonoids. The plant extract displayed significant antibacterial activity against several pathogenic bacteria. Among Gram-positive bacteria, only *Bacillus subtilis* was susceptible, with MICs ranging from 11 to 50 µg/mL. For Gram-negative bacteria, *Salmonella typhi* and *Shigella flexneri* were sensitive, with estimated MIC values of 2–7 and 8–50 µg/mL, respectively. In contrast, the antifungal activity of the plant was limited to *Microsporum canis*, with MIC values ranging from 60 to 250 µg/mL [97]. Wang et al. [98] isolated four known homoisoflavanones and evaluated their antisepsis activity against four bacterial strains and six plant pathogens at a concentration of 10 μg/mL. The results indicated that compound **25** displayed strong inhibitory effects against the growth of *Colletotrichum lagenarium*, *Alternaria brassicae*, *Verticillium dahliae*, *Exserohilum turcicum*, *Escherichia coli*, *Bacillus cereus*, and *Corynebacterium sepedonicum*. Compounds **14**, **15**, and **37** were evaluated for their antimicrobial activity. Among them, compound **37** displayed the most potent effect, forming markedly larger inhibition zones relative to the reference standards [22].

### 5.6. Other Activities

*Polygonatum* extracts and isolated flavonoids also possess additional pharmacological effects. In in vitro experiments, neuroprotective assays revealed that flavonoids isolated from *P. sibiricum* exerted significant neuroprotective effects against H_2_O_2_-induced injury in PC12 cells. Moreover, compounds **47** and **48** showed stronger activity than compound **46**, which suggests that the 5′-hydroxy moiety of the B-ring may reduce the neuroprotective effects [45]. In the present study, systematic pharmacology, molecular docking and in vitro experiments were integrated to identify the antidepressant constituents of *Polygonati Rhizoma* (PR) and clarify their mechanisms. Four flavonoids from PR were associated with 45 depression-related targets. In vitro, these flavonoids inhibited LPS-induced inflammation in BV-2 cells, improved mitochondrial function, alleviated oxidative stress, and reduced the expression of IL-1*β*, TNF-*α* and IL-6 in a dose-dependent manner. They also suppressed COX2 expression and NLRP3/caspase-1 activation, thus exerting antidepressant effects. These findings indicate that the flavonoids are key components responsible for the antidepressant activity of PR [99]. In in vivo experiments, TFPk prolonged the swimming exhaustion time, improved carbohydrate metabolism, significantly elevated GSH-Px activity and decreased MDA levels in mouse liver and skeletal muscle, and increased SOD activity in skeletal muscle. TFPk exerts anti-fatigue effects in mice, acts as an effective free radical scavenger, enhances antioxidant capacity, and attenuates tissue lipid peroxidation induced by excessive exercise [100].

**Table 2:** Pharmacological activities of flavonoids from the genus *Polygonatum*.

Pharmacology	Substances	In Vitro/In Vivo	Assay Methods/Experimental Model	Dosages	Mechanisms/Effects	Ref.
Antioxidant activity	**2**, **3**, and **27**	In vitro	DPPH	–	Compounds **2**, **3**, and **27** showed stronger antioxidant effects (IC_50_ = 4.9 ± 0.3, 3.8 ± 0.5, and 3.9 ± 0.4 μg/mL, respectively) than ascorbic acid (IC_50_ = 5.3 ± 0.6 μg/mL).	[16]
	TFPo	In vitro	DPPH	0.01–0.8 mg/mL	TFPo possess potent DPPH radical scavenging activity, and this capacity is markedly improved upon their interaction with iron salts.	[60]
	TFPs	In vitro	DPPH, ABTS	0–500 μg/mL	TFPS showed DPPH and ABTS radical scavenging effects with IC_50_ values of 27.55 and 11.47 μg/mL, and its ferrous ion-chelating activity reached an IC_50_ value of 32.26 μg/mL.	[61]
	The ethanolic extract of *P. alte-lobatum*	In vitro	DPPH	2–10 μg/mL	The extract showed DPPH radical scavenging activity, with an IC_50_ value of 9 μg/mL.	[62]
	TFPs	In vitro	DPPH, ABTS	0.1–0.5 mg/mL	When the concentration of TFPs was 0.5 mg/mL, the DPPH and ABTS radical scavenging activities peaked, achieving scavenging rates of 84.3% and 81.5%, respectively.	[63]
	FE, **25**, and **30**	In vitro	DPPH, OH, and reducing power	–	FE exhibited antioxidant activity with IC_50_ values of 0.06 ± 0.035 mg/mL against DPPH radicals and 0.68 ± 0.03 mg/mL against hydroxyl radicals, and its reducing power at 1.25 mg/mL was determined to be 0.56 ± 0.033. In addition, compounds **25** and **30** against DPPH radicals had IC_50_ values of 5.90 ± 0.150 mg/mL and 11.64 ± 0.296 mg/mL.	[64]
	TFPs	In vitro	DPPH, ABTS	0.25–3.00 mg/mL	TFPs exhibited dose-dependent DPPH and ABTS radical scavenging activities, which increased progressively as the concentration rose from 0.25 to 3.00 mg/mL.	[66]
	TFPs	In vitro	DPPH	0.1–0.5 mg/mL	When the concentration was 0.50 mg/mL, the DPPH scavenging capacity peaked at 91 %.	[67]
	TFPk	In vitro	DPPH, ABTS	1–8 mg/mL	TFPk exhibited potent scavenging activities against DPPH and ABTS free radicals, with EC_50_ values of 2.11 and 1.63 mg/mL, respectively.	[68]
	TFPk	In vitro	DPPH, ABTS	2–14 mg/mL	The scavenging rates of TFPk against DPPH and ABTS free radicals reached 82.44% and 86.22%, respectively.	[69]
Anti-diabetic activity	**25**, **26**, and **38**	In vitro	AGE formation model	0.312–0.25 μM	Compounds **25**, **26**, and **38** had notable inhibitory activity against AGE formation, with IC_50_ values of 56.30, 46.05, and 107.10 μM, respectively.	[48]
	TFPk	In vitro	*α*-Glucosidase and *α*-amylase	1–8 mg/mL	TFPk showed inhibitory activity on *α*-glucosidase and *α*-amylase, with EC_50_ values of 1.70 mg/mL and 2.69 mg/mL, respectively.	[68]
	**25**, **26**, **30**, and **71**	In vitro	IAR-20 cells	10 μM	Compounds **1**, **2**, **6**, and **70** exhibited remarkable glucose-uptake-promoting effects via the activation of the AMPK signaling pathway.	[72]
	**1**, **2**, and **6**	In vitro	Caco-2 cells	15 μM	Compounds **25**, **26**, and **30** had a stronger inhibitory effect on 25 mM glucose transport (47.5 ± 1.9 %, 41.6 ± 2.5 %, and 50.5 ± 7.6 %).	[73]
	Isorhamnetin	In vitro	Insulin-resistant (IR) HepG2 cell line	–	Isorhamnetin could significantly increase glucose consumption in insulin-resistant (IR) HepG2 cell models, upregulate the protein expression levels of PI3K and AKT1, and downregulate the protein expression levels of VEGF and mTOR.	[74]
	**25** and **26**	In vitro	HepG2 cell line	0.1–10 μM	Compound **25** (IC_50_ = 12 μM) and compound **26** (IC_50_ = 7 μM) bound to the PPARγ ligand-binding domain with fair binding affinity.	[76]
	TFPo	In vivo and In vitro	Alloxan-induced diabetic rats and *α*-amylase	50–200 mg/kg and 0.1–2%.	In vivo experiments indicated that the hypoglycemic effect of TFPo at 200 mg/kg is similar to that of acarbose 20 mg/kg and gliclazide 15 mg/kg. In vitro experiments indicated that TFPo significantly inhibited *α*-amylase activity in a dose-dependent manner.	[77]
	TFPs	In vivo and In vitro	Alloxan-induced diabetic rats and *α*-amylase	50–200 mg/kg and 0.1–2%.	In vivo experiments indicated that TFPs could significantly increase the insulin level in alloxan-induced type 2 diabetic rats compared with the control. An *α*-amylase inhibition assay in vitro showed that TFPs significantly inhibited *α*-amylase activity in a dosage-dependent manner.	[78]
Anticancer activity	**16** and **44**	In vitro	HCT-116, AGS, U-87 MG, and PC-12	–	Compound **16** showed significant and selective cytotoxicities against AGS and PC12, with IC_50_ values of 7.2 and 9.8 μM, respectively; compound **44** showed cytotoxic activities against the four tumor cell lines, with IC_50_ values in the range of 2.2–9.8 μM.	[21]
	**25** and **26**	In vitro	MCF-7 cells	10–100 μM for compound **1**, 10–80 μM for compound **2**	Compound **26** was more cytotoxic (IC_50_ = 30 μM) than compound **25** (IC_50_ = 90 μM). Compound **26** could induce Bcl-2 phosphorylation, apoptosis, and G2/M cell cycle arrest in breast tumor cells.	[35]
	**30**–**33**, and **37**	In vitro	HepG2 and A549 cells	15.625–500 μmol/L	Compounds **30**, **33** and **37** had inhibitory effects on both cancer cell lines, with a relatively strong inhibitory effect on A549. Among them, compound **37** had a notable inhibitory effect on A549, with an IC_50_ value of 17.99 ± 1.45 μmol/L	[38]
	**25**	In vitro	A549 cells	12.5–100 mg/L	Compound **25** could promote the apoptosis of A549 cells and increase the proportion of cells in G2/M via mitochondria-mediated apoptosis and the p38 MAPK pathway.	[84]
	**25**	In vitro	A549 cells	12.5–100 mg/L	Compound **25** could induce apoptosis in A549 cells by regulating the mitochondria-caspase-dependent and ER stress pathways and resulted in G2/M arrest by activating the p38/p53 signaling pathway.	[85]
	**25**–**28**	In vitro	K562, A549, HCT-15, HLE, and DHODH	0–100 μg/ mL	All compounds showed inhibitory activity against K562, A549 and HCT-15 cancer cells, with IC_50_ values of 7– 35 μg/mL. They also showed inhibitory activity against HLE, with IC_50_ values of 13.1, 70.4, 13.8, and 55.2 μg/mL, respectively. Compounds 26 and 28 showed inhibitory activity against DHODH, with IC_50_ values of 10.0 and 11.1 μg/mL, respectively.	[86]
	**80**	In vitro	MCF-7 and BT20 cell lines	0.2 mmol/L	Compound **80** could reduce aggressiveness of BC cells by suppressing HSP90-mediated CMA.	[88]
Anti-inflammatory activity	**55**, **85**, **86**, and **91**	In vitro	RAW264.7	50 μL	All compounds showed a moderate inhibitory effect against NO production, with IC_50_ values of 8.28–41.85 μmol/L and without cytotoxicity against the cells.	[23]
	**89**–**91**	In vitro	RAW264.7	–	**91** (IC_50_ = 9.89 ± 1.36 μM) showed the strongest nitric oxide inhibitory effect, followed by **89** (IC_50_ = 17.03 ± 1.28 μM), **90** (IC_50_ = 18.87 ± 1.68 μM).	[33]
	**30**–**33**, and **37**	In vitro	RAW264.7	16 μmol/L	All compounds could inhibit the release of NO. Among them, compound **31** showed good potential anti-inflammatory activity; the NO inhibition rate of **31** reached 91.16 ± 3.51 %, which was significantly higher than that of the positive control dexamethasone (64.81 ± 1.71 %).	[38]
Antibacterial activity	**14**, **15**, and **37**	In vitro	Gram-positive bacteria and Gram-negative bacteria	50 μL	Compounds **14**, **15**, and **37** exhibited noticeable antibacterial activity against non-pathogenic bacterial type strains. However, **37** showed the highest activity with the maximum inhibition zone against Gram-positive bacteria (*S. aureus* and *B. subtilis*; 15 mm), which is comparable to that achieved by the well-known antibiotic tetracycline.	[22]
	TFPv	In vitro	*Escherichia coli*, *Salmonella typhi*, *Shigella flexneri*, and *Staphylococcus aureus*	10 mg/mL	The MICs were 1.5–40, 3–6, 3–40, and 75–80 μg/mL, respectively.	[96]
	The crude methanol extract of *P. verticillatum*	In vitro	Gram-positive bacteria and Gram-negative bacteria	–	Among Gram-positive bacteria, only Bacillus subtilis was susceptible, with MIC values of 11–50 µg/mL. For Gram-negative bacteria, *Salmonella typhi* and *Shigella flexneri* were sensitive, with estimated MIC values of 2–7 µg/mL and 8–50 µg/mL, respectively.	[97]
	**25**	In vitro	Four bacterial strains and six plant pathogens	10 μg/mL	Compound **25** displayed strong inhibitory effects against the growth of *Colletotrichum lagenarium* (50 %), *Alternaria brassicae* (51.67 %), *Verticillium dahlia* (44.92 %), *Exserohilum turcicum* (58.24 %), *Escherichia coli* (13 mm), *Bacillus cereus* (9.24 mm), and *Corynebacterium sepedonicum* (10.50 mm).	[98]
Other activities	**30**, **45**, **47**, **48**, and **50**	In vitro	H_2_O_2_-induced PC12 cells	50 μM	Compounds **30**, **45**, **47**, **48**, and **50** significantly alleviated H_2_O_2_-induced damage in PC12 cells, increasing cell viability to approximately 76 %, 72 %, 74 %, 76 %, and 79 %, respectively.	[45]
	TFPk	In vivo	5-week endurance exercise in mice	100 and 200 mg/kg·d	TFPk exerted a protective effect on lipid peroxidation induced by excessive exercise in organisms, and it alleviated the oxidative damage caused by free radical lipid peroxidation.	[100]

‘–’ denotes no useful information found in the study.

## 6. Conclusions

Flavonoids are the core bioactive components of *Polygonatum*, a medicinal and edible genus with high research value and application potential, and their chemical composition, biosynthesis pathways, and pharmacological activities have been extensively explored in recent years. *Polygonatum* is rich in diverse flavonoids, with more than 90 compounds identified, belonging to eight subclasses: homoisoflavanones, flavones, isoflavones, chalcones, flavanones, isoflavanones, flavonols, and pterocarpans. Homoisoflavanones, as the most characteristic subclass with 54 isolated compounds, are distinguished by their unique C6-C4-C6 skeleton, while flavones in this genus mainly exist in the form of C-glycosides, which enhance their stability and bioavailability, showing obvious species-specific distribution characteristics among different *Polygonatum* species.

The flavonoid biosynthesis pathway in *Polygonatum* is initiated with phenylalanine as the starting substrate. Phenylalanine is first converted to cinnamic acid by PAL and is then hydroxylated to *p*-coumaric acid via C4H. 4CL further activates *p*-coumaric acid by converting it to *p*-coumaroyl-CoA, which condenses with malonyl-CoA under the catalysis of CHS to yield naringenin chalcone. CHI mediates the stereospecific isomerization of the chalcone into a flavanone, which serves as the central intermediate for downstream flavonoid derivatives through the catalysis of a series of enzymes. Notably, the specific enzyme PcAS1 in *Polygonatum* specifically catalyzes the 3-benzylation migration of naringenin, thereby producing the 3-benzylchroman-4-one skeleton unique to homoisoflavonoids. Currently, the phenylpropanoid pathway upstream of flavonoid biosynthesis in *Polygonatum* has been thoroughly studied. However, the specific biosynthetic steps from flavanones to homoisoflavanones have not been fully clarified and validated. Furthermore, the cytochrome P450s and glycosyltransferases involved in this metabolic pathway remain to be further elucidated.

Meanwhile, *Polygonatum* flavonoids exhibit comprehensive and significant antioxidant, anti-diabetic, anticancer, anti-inflammatory, and antibacterial bioactivities, which are closely related to their structural modifications, such as hydroxyl and glycosylation. Their advantages of low toxicity and high biocompatibility make them ideal candidates for natural drugs and functional foods, fully supporting the traditional medicinal value of *Polygonatum* and providing a basis for its modern medicinal development. Meanwhile, the structure–activity relationship revealed that homoisoflavanones bearing dihydroxylated B-rings may exert stronger antioxidant activity, whereas the 5′-hydroxy substituent on the B-ring may reduce neuroprotective effects. Meanwhile, the homoisoflavonoid (3R)-5,7-dihydroxy-6-methyl-8-methoxy-3-(4′-hydroxybenzyl)-chroman-4-one (**25**) displays a wide spectrum of bioactivities, such as antioxidant, anti-diabetic, anticancer and antibacterial activities, suggesting its strong potential for the future development of health-promoting pharmaceuticals. Nevertheless, studies on the antioxidant and anticancer activities of flavonoids from this genus are currently limited to in vitro experimental verification, while their underlying pharmacological mechanisms and in vivo investigations remain largely unexplored. Therefore, further exploration of the structure–activity relationship is warranted. This will lay a foundation for the in-depth development and utilization of *Polygonatum* flavonoids and promote systematic research on this genus.

## Data Availability

The data presented in this study are available on request from the corresponding author.

## Abbreviations

The following abbreviations are used in this manuscript:

TFPo	Total flavonoids of *P. odoratum*	PC12	Rat pheochromocytoma cells
TFPs	Total flavonoids of *P. sibiricum*	PCL	*P. cyrtonema* lectin
TFPk	Total flavonoids of *P. kingianum*	PI3K	Phosphatidylinositol-3-kinase
TFPv	Total flavonoids of *P. verticillatum*	AMPK	Adenosine monophosphate-activated kinase
TCM	Traditional Chinese medicine	BG	Blood glucose
Glc	*β*-D-glucopyranosyl	AMP	Adenosine monophosphate
Xyl	Xylopyranoside	3T3-L1	3T3-L1 mouse embryonic fibroblast cells
Gal	Galactopyranose	ER	Endoplasmic reticulum
Rha	*α*-L-rhamnopyranose	PAL	Phenylalanine ammonialyase
GlcA	*β*-D-Glucopyranuronic acid	HCT-116	Human colon tumor 116
C4H	Cinnamate 4-hydroxylase	AGS	Adenocarcinoma gastric strain
4CL	4-Coumarate-CoA ligase	U-87 MG	Uppsala 87 malignant glioma
CHS	Chalcone synthase	MIC	Minimum inhibitory concentration
CHI	Chalcone isomerase	MCF-7	Michigan Cancer Foundation-7
FNS	Flavone synthase	BT20	Breast tumor 20
IFS	Isoflavone synthase	HepG2	Hepatoma G2
FLS	Flavonol synthase	A549	Human lung adenocarcinoma A549 cell line
PcAS1	*Polygonatum cyrtonema* cyclase 1	K562	K 562 Human chronic myeloid leukemia cells
HID	2-Hydroxyisoflavanone dehydratase	HCT-15	Human colon tumor 15
Aβ	Amyloid *β*-peptide	HLE	Human hepatocellular carcinoma cell line
Ac	Acetyl	DHODH	Dihydroorotate dehydrogenase
RAW264.7	Murine macrophage cells	IAR-20	Rat liver epithelial cells
DPPH	2,2-Diphenyl-1-picrylhydrazyl	Caco-2 cells	Colon carcinoma clone 2
ABTS	2,2′-Azino-bis(3-ethylbenzothiazoline-6-sulfonic acid)	Glut2	Glucose transporter 2
IC_50_	50% inhibitory concentration	IR	Insulin-resistant
EC_50_	Effective concentration 50%	AGEs	Advanced glycation end products
HSCCC	High-speed counter-current chromatography	PPARγ	Peroxisome proliferator-activated receptor gamma
iNOS	Inducible nitric-oxide synthase	MDA	Malondialdehyde
LPS	Lipopolysaccharide	SOD	Superoxide oxide dismutase
NO	Nitric oxide	GSH-Px	Glutathione peroxidase
